# Supplementary material for: Iterative improvement in the automatic modular design of robot swarms
Source: PeerJ Comput Sci. 2020 Dec 7;6:e322. doi: 10.7717/peerj-cs.322 (PMC7924708; doi:10.7717/peerj-cs.322)
Supplement: Supplemental Information 3 [file peerj-cs-06-322-s003.zip › argos3/doc/api/standalone/a00416_source.html]

ARGoS: plugins/robots/e-puck/simulator/physx\_epuck\_model.cpp Source File


- Main Page
- Related Pages
- Namespaces
- Classes
- Files

- File List
- File Members

# plugins/robots/e-puck/simulator/physx\_epuck\_model.cpp

Go to the documentation of this file.

```
00001 
00007 #include "physx_epuck_model.h"
00008 #include <argos3/plugins/robots/e-puck/simulator/epuck_entity.h>
00009 
00010 namespace argos {
00011 
00012    /****************************************/
00013    /****************************************/
00014    
00015    static const Real EPUCK_INTERWHEEL_DISTANCE = 0.053f;
00016    static const Real EPUCK_WHEEL_RADIUS        = 0.0205f;
00017    static const Real EPUCK_WHEEL_THICKNESS     = 0.01f;
00018    static const Real EPUCK_WHEEL_MASS          = 0.05f;
00019 
00020    static const Real EPUCK_CHASSIS_LENGTH      = 0.055f;
00021    static const Real EPUCK_CHASSIS_WIDTH       = (EPUCK_INTERWHEEL_DISTANCE - EPUCK_WHEEL_THICKNESS) * 0.9f;
00022    static const Real EPUCK_CHASSIS_HEIGHT      = EPUCK_WHEEL_RADIUS * 2.0f;
00023    static const Real EPUCK_CHASSIS_ELEVATION   = 0.005f;
00024    static const Real EPUCK_CHASSIS_MASS        = 0.3f;
00025 
00026    static const Real EPUCK_BOARD_RADIUS        = 0.035f;
00027    static const Real EPUCK_BOARD_HEIGHT        = 0.03f;
00028 
00029    enum EPUCK_WHEELS {
00030       EPUCK_LEFT_WHEEL = 0,
00031       EPUCK_RIGHT_WHEEL = 1
00032    };
00033 
00034    /****************************************/
00035    /****************************************/
00036    
00037    CPhysXEPuckModel::CPhysXEPuckModel(CPhysXEngine& c_engine,
00038                                       CEPuckEntity& c_entity) :
00039       CPhysXMultiBodyObjectModel(c_engine, c_entity, 3),
00040       m_fCurrentWheelVelocity(c_entity.GetWheeledEntity().GetWheelVelocities()),
00041       m_cDiffDrive(*this,
00042                    c_engine,
00043                    EPUCK_INTERWHEEL_DISTANCE,
00044                    EPUCK_WHEEL_RADIUS,
00045                    EPUCK_WHEEL_THICKNESS,
00046                    EPUCK_WHEEL_MASS,
00047                    physx::PxVec3(EPUCK_CHASSIS_LENGTH,
00048                                  EPUCK_CHASSIS_WIDTH,
00049                                  EPUCK_CHASSIS_HEIGHT),
00050                    EPUCK_CHASSIS_ELEVATION,
00051                    EPUCK_CHASSIS_MASS) {
00052       /* Get position and orientation in this engine's representation */
00053       physx::PxTransform cBodyTrans;
00054       CVector3ToPxVec3(GetEmbodiedEntity().GetOriginAnchor().Position, cBodyTrans.p);
00055       CQuaternionToPxQuat(GetEmbodiedEntity().GetOriginAnchor().Orientation, cBodyTrans.q);
00056       /*
00057        * Top board
00058        */
00059       /* Create cylinder geometry */
00060       physx::PxConvexMeshGeometry* pcBoardGeometry =
00061          CreateCylinderGeometry(c_engine,
00062                                 EPUCK_BOARD_RADIUS,
00063                                 EPUCK_BOARD_HEIGHT);
00064       /* Create the shape and attach it to the differential drive component */
00065       physx::PxShape* pcBoardShape =
00066          m_cDiffDrive.GetMainBodyActor().createShape(*pcBoardGeometry,
00067                                                      GetPhysXEngine().GetDefaultMaterial());
00068       pcBoardShape->userData = this;
00069       /* Set offset transformation for the board */
00070       pcBoardShape->setLocalPose(
00071          physx::PxTransform(0.0f,
00072                             0.0f,
00073                             EPUCK_CHASSIS_ELEVATION +
00074                             EPUCK_CHASSIS_HEIGHT +
00075                             EPUCK_BOARD_HEIGHT * 0.5f));
00076       /* Place the differential drive component in its initial position */
00077       m_cDiffDrive.SetGlobalPose(cBodyTrans);
00078       /* Calculate the bounding box */
00079       CalculateBoundingBox();
00080       /* Cleanup */
00081       delete pcBoardGeometry;
00082       /* Register the origin anchor update method */
00083       RegisterAnchorMethod(GetEmbodiedEntity().GetOriginAnchor(),
00084                            &CPhysXEPuckModel::UpdateOriginAnchor);
00085    }
00086 
00087    /****************************************/
00088    /****************************************/
00089 
00090    void CPhysXEPuckModel::Reset() {
00091       /* Reset bodies */
00092       CPhysXMultiBodyObjectModel::Reset();
00093       /* Zero wheel speeds */
00094       m_cDiffDrive.SetTargetWheelLinearVelocity(0.0f, 0.0f);
00095    }
00096 
00097    /****************************************/
00098    /****************************************/
00099 
00100    void CPhysXEPuckModel::UpdateFromEntityStatus() {
00101       /* Do we want to move? */
00102       if((m_fCurrentWheelVelocity[EPUCK_LEFT_WHEEL] != 0.0f) ||
00103          (m_fCurrentWheelVelocity[EPUCK_RIGHT_WHEEL] != 0.0f)) {
00104          m_cDiffDrive.SetTargetWheelLinearVelocity
00105             (m_fCurrentWheelVelocity[EPUCK_LEFT_WHEEL],
00106              m_fCurrentWheelVelocity[EPUCK_RIGHT_WHEEL]);
00107       }
00108       else {
00109          /* No, we don't want to move - zero all speeds */
00110          m_cDiffDrive.SetTargetWheelLinearVelocity(0.0f, 0.0f);
00111       }
00112    }
00113 
00114    /****************************************/
00115    /****************************************/
00116 
00117    void CPhysXEPuckModel::UpdateOriginAnchor(SAnchor& s_anchor) {
00118       /* Get transform of the ARGoS origin anchor */
00119       physx::PxTransform cBodyTrans =
00120          m_cDiffDrive.GetMainBodyActor().getGlobalPose() *
00121          m_cDiffDrive.GetMainBodyOffset().getInverse();
00122       /* Set object position into ARGoS space */
00123       PxVec3ToCVector3(cBodyTrans.p, s_anchor.Position);
00124       /* Set object orientation into ARGoS space */
00125       PxQuatToCQuaternion(cBodyTrans.q, s_anchor.Orientation);
00126       physx::PxReal fLeftAngle;
00127       physx::PxReal fRightAngle;
00128       m_cDiffDrive.GetCurrentWheelAngles(fLeftAngle, fRightAngle);
00129       DEBUG("Current wheel rotation: %f, %f\n", fLeftAngle, fRightAngle);
00130       physx::PxReal fLeftVelocity;
00131       physx::PxReal fRightVelocity;
00132       m_cDiffDrive.GetCurrentWheelLinearVelocity(fLeftVelocity, fRightVelocity);
00133       DEBUG("Current wheel speed: %f, %f\n", fLeftVelocity, fRightVelocity);
00134       physx::PxReal fLeftTargetVelocity;
00135       physx::PxReal fRightTargetVelocity;
00136       m_cDiffDrive.GetTargetWheelLinearVelocity(fLeftTargetVelocity, fRightTargetVelocity);
00137       DEBUG("Target wheel speed: %f, %f\n\n", fLeftTargetVelocity, fRightTargetVelocity);
00138    }
00139 
00140    /****************************************/
00141    /****************************************/
00142 
00143    REGISTER_STANDARD_PHYSX_OPERATIONS_ON_ENTITY(CEPuckEntity, CPhysXEPuckModel);
00144 
00145    /****************************************/
00146    /****************************************/
00147 
00148 }
```

---

Generated on 10 Jul 2018 for ARGoS by 
 1.6.1
